# Characteristics and Clinical Outcomes of BRCA Germline Mutation Carriers with Advanced Breast Cancer Treated with PARP (Poly ADP-Ribose Polymerase) Inhibitors: A Single-Institution Experience †

**DOI:** 10.3390/cancers18081258

**Published:** 2026-04-16

**Authors:** Fatma Nihan Akkoc Mustafayev, Elena Fountzilas, Mark F. Munsell, Rachel M. Layman, Clinton Yam, Angelica M. Gutierrez, Constance T. Albarracin, Zamal Ahmed, Katharina Schlacher, John A. Tainer, Banu K. Arun

**Affiliations:** 1 Department of Breast Medical Oncology, The University of Texas MD Anderson Cancer Center, Houston, TX 77030, USA; nihanakkoc88@gmail.com (F.N.A.M.);; 2 Department of Medical Oncology, St Luke’s Clinic, 55236 Thessaloniki, Greece; 3 Department of Biostatistics, The University of Texas MD Anderson Cancer Center, Houston, TX 77030, USA; 4 Department of Pathology, The University of Texas MD Anderson Cancer Center, Houston, TX 77030, USA; 5 Department of Molecular and Cellular Oncology, The University of Texas MD Anderson Cancer Center, Houston, TX 77030, USA; 6 Department of Cancer Biology, The University of Texas MD Anderson Cancer Center, Houston, TX 77030, USA

**Keywords:** germline mutation, *BRCA1*, *BRCA2*, breast cancer, real-world, PARP inhibitors

## Abstract

**Background/Objectives**: Several trials have highlighted the importance of PARP inhibitors (PARPi) in the treatment of *BRCA*-associated breast cancers (BC), initiating changes in practice. However, data on the real-life outcomes of PARPi therapy is limited. In this study, we characterized the clinical characteristics and outcomes of patients with advanced BC and germline *BRCA* pathogenic variants (PVs) who received PARPi therapy. **Methods**: We conducted a retrospective single-institution cohort study of patients with advanced BC and germline *BRCA1/2* PVs treated with PARPi. Outcomes included objective response rate (ORR), progression-free survival (PFS), and overall survival (OS). Survival was estimated using Kaplan–Meier methods, and prognostic factors were evaluated using Cox regression analysis. **Results**: Of the 107 patients treated with PARPi, 48 (44.9%) and 59 (55.1%) had *BRCA1* and *BRCA2* PVs, respectively. Ninety-seven patients (90.7%) had invasive ductal carcinoma and 42 (39.3%) had triple-negative BC. Nineteen (17.8%) patients had de novo metastatic BC. Sixty-two (57.9%) patients received at least one line of systemic therapy before PARPi; 24 (22.4%) patients received prior platinum. ORR was 62.6%, and the median duration of response (DoR) was 7 months (range, 2.1–96.2). The median PFS was 9 months (95% CI, 6.9–10.5) and median OS was 25.8 months (95% CI, 18.7–31.5). In multivariable models for PFS, bone metastases (HR = 2.25; 95% CI, 1.40–3.61; *p* = 0.0008) and lung metastases (HR = 2.40; 95% CI, 1.45–3.98; *p* = 0.0007) were independently associated with increased risk of progression or death. In multivariable models for OS, brain metastases (HR = 3.54; 95% CI, 1.59–7.90; *p* = 0.0020), bone metastases (HR = 2.22; 95% CI, 1.27–3.88; *p* = 0.0050), and lung metastases (HR = 2.38; 95% CI, 1.38–4.11; *p* = 0.0018), were independently associated with increased risk of death. **Conclusions**: The clinical outcomes of our real-world patients are similar to those reported in previous clinical trials. In addition, metastatic site distribution was independently prognostic for survival outcomes and may support baseline risk stratification at the time of PARPi initiation. Further studies of predictive markers of response and resistance, as well as sequencing with platinums and combinations with other targeted agents, are needed to optimize the benefits of PARPi in this patient population.

## Simple Summary

Pathogenic germline variants of *BRCA1* and *BRCA2* account for the majority of hereditary breast cancer and are increasingly used to evaluate eligibility for PARP inhibitor (PARPi) therapy of *BRCA*-associated BC. However, there has been limited literature on the outcomes of PARPi therapy. In this study we found an overall response rate of 62.6% and a median progression-free survival of 9 months, supporting their integration into standard care and reinforcing the importance of routine germline *BRCA* testing to enable personalized treatment. In multivariable analyses, metastatic site distribution remained independently prognostic, with lung and bone metastases associated with increased risk of progression or death and brain, lung, and bone metastases associated with increased risk of death, identifying a higher-risk phenotype at PARPi initiation. These findings support incorporating metastatic sites into baseline risk stratification and highlight the need for larger multi-institutional studies to validate prognostic factors and to advance research on predictive biomarkers and combination strategies to optimize PARPi benefit.

## 1. Introduction

Breast cancer (BC) is the most commonly diagnosed cancer among women in the United States, accounting for approximately one third of all new female cancer diagnoses, with an estimated 321,910 new cases and 42,140 deaths projected in 2026 [1]. It remains a significant global health concern. While most cases are sporadic, approximately 5–10% are hereditary, with pathogenic germline variants in *BRCA1* and *BRCA2* accounting for the majority [2,3].

*BRCA1*/*BRCA2* genes function as tumor suppressors and play crucial roles in the regulation of double-strand break repair, genomic stability, and homologous recombination in response to DNA damage [4,5,6]. Poly (ADP-ribose) polymerase (PARP) enzymes are key components of the base excision repair pathway and are essential for the repair of DNA single-strand breaks [7,8]. The inhibition of PARP enzymes leads to single-strand breaks persisting, resulting in stalled replication forks and double-strand breaks [9]. In tumor cells with homologous recombination deficiency, treatment with a PARP inhibitor (PARPi) renders the cells unable to repair DNA damage through the base excision repair or homologous recombination pathways, leading to cell death [10,11,12]. Furthermore, it has been shown that PARPi trap the PARP enzymes on DNA, rendering the complex more lethal than single-strand breaks [13,14].

Currently, only two PARPi (olaparib and talazoparib) have been approved by the US Food and Drug Administration (FDA) as single agents for HER2-negative and germline *BRCA1*/*BRCA2*-mutated advanced BC. The OlympiAD study was a phase III randomized trial of olaparib vs. treating physician’s choice of chemotherapy (TPC). Olaparib was associated with improved progression-free survival (PFS) compared to TPC (7.0 vs. 4.2 months, respectively; hazard ratio [HR], 0.58; 95% CI, 0.43–0.80; *p* < 0.001). In addition, olaparib was associated with a higher objective response rate (ORR) compared to chemotherapy (59.9% vs. 28.8%) [15]. The phase III EMBRACA study demonstrated that compared to TPC, single-agent talazoparib was associated with a significantly increased PFS (8.6 months vs. 5.6 months; HR, 0.54; 95% CI, 0.41–0.71; *p* < 0.001) and ORR (62.6% vs. 27.2%) [16].

PARPi are now widely used in clinical practice; however, real-world studies have primarily focused on treatment outcomes, with limited evaluation of factors influencing response and survival. We present the real-world clinical outcomes of patients with advanced BC and *BRCA* PVs who received PARPi therapy. The objective of this study was to define patient demographics, clinical characteristics, and clinical outcomes, namely PFS, overall survival (OS), and ORR, and to evaluate clinically relevant prognostic factors at treatment initiation to better inform risk stratification in routine practice.

## 2. Materials and Methods

### 2.1. Patient Population and Data Collection

We searched the prospective research database of The University of Texas MD Anderson Cancer Center Breast Medical Oncology for patients with germline *BRCA1*/*BRCA2* PVs who were treated with PARPi from 1 June 2008, through 31 December 2022, for metastatic or recurrent disease. Patients with *BRCA1*/*BRCA2* somatic mutations or genetic variants of unknown significance were excluded from the study. Patients with missing key covariates or survival follow-up were excluded from inferential analyses.

This study was approved by The University of Texas MD Anderson Cancer Center’s Institutional Review Board (protocol LAB03-0479 and RCR06-0561). This retrospective analysis of prospectively collected data included age at diagnosis, race, family history of BC and/or ovarian cancer, menopausal status, prophylactic surgery information, patient demographics, tumor characteristics including histological type, receptor status, body mass index (BMI), type of systemic therapy received, recurrence, and survival information.

### 2.2. Pathologic Assessment

All pathologic specimens were assessed by dedicated breast pathologists at MD Anderson. Invasive carcinoma was confirmed using initial core biopsy specimens. The initial clinical stage and pathologic stage of all patients were obtained from the patients’ medical records. Positive estrogen receptor and progesterone receptor status was defined as nuclear staining in ≥1% of cells on immunohistochemistry. HER2/neu-positive status was defined as immunohistochemistry 3+ and/or presence of gene amplification as determined by fluorescence in situ hybridization per ASCO/CAP definitions at the time of diagnosis.

### 2.3. Statistical Analysis and Outcome Measures

The primary outcomes assessed in this study were PFS, OS, and ORR. The demographic and clinical characteristics were summarized by descriptive statistics. We estimated the ORR and disease control rate with 95% exact binomial confidence intervals. Tumor response was assessed retrospectively using available imaging reports, including computed tomography (CT) and/or positron emission tomography–CT and clinician documentation; when formal RECIST assessments were available, they were used. In the absence of formal RECIST assessments, response was classified based on the treating physician’s clinical assessment, corroborated by radiologic findings contained within the medical chart. Objective response was defined as complete response or partial response. Disease control was defined as an objective response and/or stable disease.

The product-limit estimator of Kaplan and Meier was used to estimate the median OS and PFS. The Cox proportional hazards regression model was used to estimate the impact of potential risk factors for OS and PFS in univariate analyses. Those potential risk factors with a *p*-value < 0.25 were considered in a full model, and then backward elimination was used to remove factors with a *p*-value > 0.05 until all remaining factors in the model had a *p*-value < 0.05. The final multivariable model for PFS included two variables (bone metastases and lung metastases), and the final multivariable model for OS included three variables (bone metastases, brain metastases, and lung metastases). We followed the recommendations of Peduzzi et al. (1995) when building our models [17]. For categorical variables, the log(-log) of the Kaplan–Meier survival curves were plotted against the log of survival time to assess the suitability of the proportional hazard assumption. PFS was defined as the time from therapy initiation of PARPi to disease progression or death, whichever occurred first. OS was defined as the time from therapy initiation of PARPi to death of any cause. A *p* value less than 0.05 indicated statistical significance. All statistical analyses were performed using SAS 9.4 for Windows (© 2020 by SAS Institute Inc., Cary, NC, USA) and figures were created using R 4.2.1 (© 2022 by the R Foundation for Statistical Computing, Vienna, Austria).

## 3. Results

### 3.1. Patient Demographics and Clinical Characteristics

A total of 107 patients were included in our study. The median age at diagnosis was 38 years (range, 23–73); most were female (98.1%) and most were White (71%). *BRCA1* and *BRCA2* germline PVs were identified in 48 (44.9%) and 59 (55.1%) patients, respectively. A family history of BC and/or ovarian cancer was reported in 83.2% of patients. Overall, 97 patients (90.7%) had invasive ductal carcinoma and 42 (39.3%) had triple-negative BC. Nineteen (17.8%) patients had de novo metastatic BC (stage IV). Among patients with metastatic disease (*n* = 102) prior to PARPi therapy, the most frequent sites of involvement were bone (*n* = 49, 48.0%), liver (*n* = 35, 34.3%), and lung (*n* = 30, 29.4%). Brain metastases were less common, occurring in 8.8% of patients.

Prior to receiving treatment with PARPi, 62 patients (57.9%) had received one or more lines of systemic therapy (which may have included chemotherapy, hormonal therapy, targeted therapy, and/or immunotherapy); 24 patients (22.4%) had previously received platinum in a neoadjuvant, adjuvant, or metastatic setting. Fifty-two (48.6%) patients received olaparib, 31 (29.0%) received talazoparib, 21 (19.6%) received veliparib, and 3 (2.8%) received other PARPi treatment. Forty-eight (44.9%) patients received PARPi as the standard-of-practice treatment. Patient demographics and key clinical characteristics are summarized in Table 1; additional baseline characteristics including surgical history, menopausal status, prior treatment details, and platinum exposure by treatment setting, are provided in Supplementary Table S1.

### 3.2. Clinical Outcomes

A total of 96 patients received at least two cycles (28 days/cycle) of PARPi treatment, and 91 of them were eligible for an assessment of response (Table 2). The ORR was 62.6% (95% CI, 51.9–72.6%), and the median DoR was 7 months (range, 2.1–96.2 months). In platinum-naïve patients, the ORR was 70.4% (95% CI, 58.4–80.7%), and the median DoR was 7.4 months (range, 2.1–96.2 months). In patients who received PARPi as first-line therapy, the ORR was 72.1% (95% CI, 56.3–84.7%), and the median DoR was 7.1 months (range, 2.3–32.1 months).

Patient characteristics by response status are summarized in Supplementary Table S2. Responders (those achieving complete or partial response) and non-responders (those with stable or progressive disease) did not differ significantly in baseline demographic and clinical features including age, BMI, race, BRCA mutation type, hormone-receptor status, or PARPi drug type. However, prior platinum exposure was significantly more common among non-responders (38.2% vs. 10.7%, *p* = 0.0031), and prior platinum-based neoadjuvant or adjuvant therapy was also more frequent in non-responders (20.6% vs. 5.3%, *p* = 0.0362).

**Figure 1:**
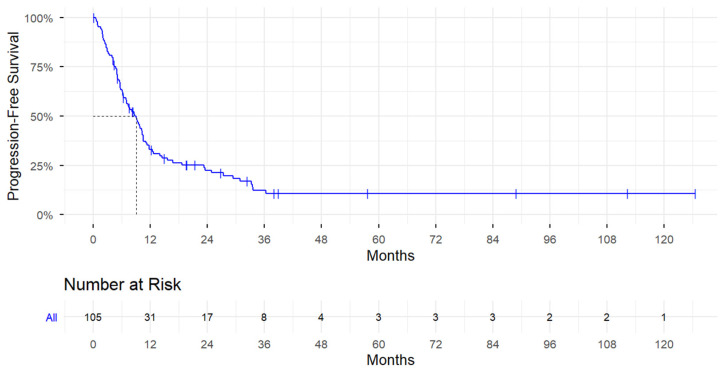
Kaplan–Meier Curves for Progression-Free Survival Among All Patients.

Among all patients, the median PFS was 9 months (95% CI, 6.9–10.5), and 84 (78.5%) patients experienced disease progression or death (Figure 1). Univariate associations with PFS are provided in Table 3. BMI was not significantly associated with PFS. Patients who had received previous systemic therapy for advanced BC had an increased risk of progression or death compared to those without previous systemic therapy for advanced BC (HR = 1.65; 95% CI, 1.06–2.56; *p* = 0.0274) (Supplementary Figure S1). No statistically significant differences in PFS were observed according to PARPi drug type. Patients receiving PARPi therapy in a research setting were at an increased risk of progression or death compared to patients receiving PARPi therapy as the standard-of-practice (HR = 1.59; 95% CI 1.00–2.52; *p* = 0.0478) (Supplementary Figure S2). Patients with bone metastases (HR = 1.72; 95% CI, 1.11–2.65; *p* = 0.0144) (Supplementary Figure S3), lung metastases (HR = 1.76; 95% CI, 1.11–2.78; *p* = 0.0166) (Supplementary Figure S4), and other distant metastatic sites (HR = 1.60; 95% CI, 1.01–2.53; *p* = 0.0466) were also at significantly increased risk of disease progression or death.

**Figure 2:**
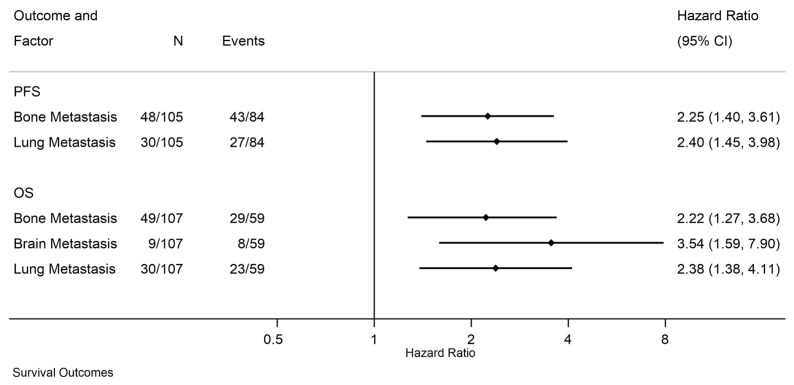
Forest Plot of Multivariable Cox Models for Progression-Free and Overall Survival.

On multivariate analysis, only lung metastases (HR = 2.40; 95% CI, 1.45–3.98; *p* = 0.0007) and bone metastases (HR = 2.25; 95% CI, 1.40–3.61; *p* = 0.0008) remained independently associated with an increased risk of progression or death (Figure 2).

**Figure 3:**
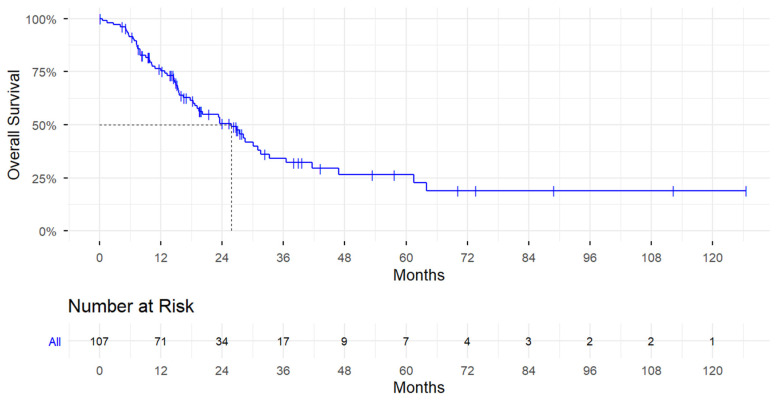
Kaplan–Meier Curves for Overall Survival Among All Patients.

By the end of the data collection period, 59 (55.1%) patients died from BC (Table 1). Eight (7.5%) patients were alive with no evidence of disease at last follow-up. The median OS was 25.8 months (95% CI, 18.7–31.5) (Figure 3). Univariate associations with OS are provided in Table 4. The median OS was numerically higher in patients who received PARPi as a first-line treatment (30.9 months) compared to those who received it as a subsequent treatment (18.7 months); however, the difference was not statistically significant (HR = 1.49; 95% CI, 0.88–2.53; *p* = 0.1352). Patients with triple-negative BC had twice the risk of death compared to those with estrogen receptor-positive and/or progesterone receptor-positive disease (HR = 1.97; 95% CI, 1.17–3.31; *p* = 0.0107) (Supplementary Figure S5). No statistically significant differences in OS were observed according to PARPi drug type. Patients with brain metastases had over twice the risk of death (HR = 2.48; 95% CI, 1.16–5.32; *p* = 0.0196) (Supplementary Figure S6), and patients with lung metastases had nearly twice the risk of death compared with those without lung metastases (HR = 1.91; 95% CI, 1.13–3.23; *p* = 0.0159) (Supplementary Figure S7).

In multivariate analysis, only brain (HR = 3.54; 95% CI, 1.59–7.90; *p* = 0.0020), bone metastases (HR = 2.22; 95% CI, 1.27–3.68; *p* = 0.0050), and lung (HR = 2.38; 95% CI, 1.38–4.11; *p* = 0.0018) remained independently associated with an increased risk of death (Figure 2).

## 4. Discussion

In this study, we evaluated the clinical outcomes of patients with advanced BC and germline *BRCA1*/*BRCA2* PVs treated with PARPi. Our findings confirm that the benefits observed in controlled trials are applicable to routine clinical practice, supporting the effectiveness of these agents in real-world settings. Beyond demonstrating trial-consistent outcomes, this study highlights metastatic site distribution as a strong independent prognostic factor.

Some demographic and clinical characteristics of the patients in this study differ from those in other studies. One important difference is the lower prevalence of triple-negative BC in this study (39.3%) compared to those in the OlympiAD and EMBRACA trials (49.8% and 45.3%, respectively). The distribution of germline *BRCA1*/*BRCA2* PVs varied as well, with this study showing a distribution (*BRCA1* = 44.9%, *BRCA2* = 55.1%) more similar to that of the EMBRACA trial (*BRCA1* = 46.3%, *BRCA2* = 54.7%) than that of the OlympiAD trial (*BRCA1* = 57.1%, *BRCA2* = 41%) [15,16]. Additionally, more patients received PARPi as a first-line treatment in this study (42.1%) compared to the previous trials (38.7% in EMBRACA and 33.2% in OlympiAD) [15,16]. However, despite these differences, the median PFS (9 months) and ORR (62.6%) observed in this study were strikingly consistent with the results from the talazoparib arm in the EMBRACA study (median PFS, 8.6 months; ORR, 62.6%) [16], suggesting that PARPi is an effective treatment option for these patients. This consistency in outcomes across various patient populations underscores the robustness of PARPi therapy in this context. Similarly, in the OlympiAD study, patients who received olaparib had a median PFS of 7 months and an ORR of 59.9% [15]. Notably, a meta-analysis of clinical trials reporting pooled response rates of PARPi in metastatic BC showed a weighted mean ORR of 45% (range, 0–78%) [18], indicating that real-world data in this study yielded more favorable response rates.

Importantly, metastatic disease distribution and metastatic site-specific outcomes provide clinically actionable prognostic information. In this study, bone and lung metastases were independently associated with a higher risk of progression, and brain, bone, and lung metastases were independently associated with a higher risk of death. These findings reinforce that visceral and central nervous system involvement identify a higher-risk subgroup even in the setting of PARPi therapy. Consistent with this, subgroup analyses from OlympiAD further support clinically important heterogeneity among patient subgroups, including those with visceral and central nervous system involvement, highlighting the prognostic relevance of metastatic burden and sites in PARPi-treated populations [19].

Consistent with findings of previous studies, the subgroup analysis of this study revealed a higher ORR among patients who received PARPi as a first-line therapy (72.1%) and for patients who had not received platinum-based therapies (70.4%). Median PFS was longer in patients who received PARPi as a first-line therapy (11.3 months) and was numerically longer among patients without prior platinum exposure (9.2 months); however, prior platinum exposure was not a statistically significant predictor of PFS in univariate analysis. Notably, in the response-evaluable cohort, prior platinum exposure was more common among non-responders than responders, consistent with a lower likelihood of objective response after platinum-based therapy. This observation is consistent with the known overlap in sensitivity and resistance mechanisms between these two drug classes, both of which exploit homologous recombination deficiency for their cytotoxic activity. The LATER-BC retrospective multicentric study similarly demonstrated that sensitivity and resistance to PARPi and platinum-based chemotherapy partially overlap in patients with germline *BRCA* PVs and advanced BC, underscoring the clinical relevance of treatment sequencing [20]. These findings suggest that PARPi should be prioritized before platinum-based therapy where clinically feasible, in order to preserve homologous recombination deficiency and maximize PARPi benefit. In a phase III study of *BRCA*-associated BC, a statistically significant improvement in PFS was observed in patients taking a combination of veliparib with carboplatin plus paclitaxel compared to patients taking the chemotherapy doublet alone, and the median PFS (14.5 months) was longer in the veliparib arm compared to that seen with PARPi in studies of OlympiAD and EMBRACA [15,16]. Additionally, similar to the present study, a higher ORR of 75.8% was observed in patients in the veliparib arm compared to those in other studies [21].

To the best of our knowledge, this study is the first to assess the association between BMI and outcomes in patients with advanced BC treated with PARPi. Numerous studies have indicated a strong association between obesity and BC, showing that obesity adversely impacts both BC incidence and outcomes [22,23,24]. However, data specifically addressing the impact of BMI on clinical outcomes in this setting remain scarce. Franzoi et al. observed no significant difference in PFS according to BMI (*p* = 0.07) in a pooled analysis of the MONARCH 2 and 3 studies, comparing abemaciclib plus endocrine therapy to endocrine therapy alone in patients with advanced BC [25]. Similarly, a retrospective study evaluating the efficacy and toxicity of CDK4/6 inhibitors in metastatic BC reported that overweight patients were likely to have a higher 12-month PFS compared to patients with a normal weight and patients with obesity (72.2%, 52.9%, and 56.5%, respectively). Notably, these differences were not statistically significant (*p* = 0.054) [26]. Consistent with prior reports, this study found no statistically significant association between BMI and PFS. When patients with a BMI < 25 kg/m^2^ were used as the reference group, neither overweight (HR = 0.92; 95% CI, 0.55–1.54; *p* = 0.7548) nor obese patients (HR = 0.76; 95% CI, 0.44–1.30; *p* = 0.3112) demonstrated a significant difference in PFS. BMI was not retained as an independent predictor in the multivariable model, and no BMI-associated benefit was observed for OS. Given the modest sample size and the potential for residual confounding, this finding should be interpreted with caution and considered exploratory. Future studies incorporating body composition parameters (e.g., sarcopenia and visceral adiposity) may clarify the impact of patient factors on the efficacy of PARPi.

A striking finding of our study was a longer median OS compared to those of the OlympiAD [27,28] and EMBRACA [29] studies (25.8 months, 19.3 months, and 19.3 months, respectively). This extended OS could be due to various factors, including patient selection, treatment sequencing, other treatment strategies, or confounding variables. Real-world studies have also demonstrated variability in OS outcomes. The LUCY study [30], which evaluated olaparib in a real-world setting, reported a median OS of 24.94 months, whereas a separate analysis of talazoparib-treated patients reported a shorter median OS of 11.6 months [31]. Although a subgroup analysis in the OlympiAD trial did not show a difference in survival between cohorts of patients with triple-negative BC and hormone receptor-positive BC, there was a potentially significant benefit for olaparib in patients who had not received chemotherapy for metastatic disease [15,27,28]. In the EMBRACA study, no significant OS benefit was noted between clinically relevant subgroups [29]. In contrast to these studies, our study suggested improved OS benefit among patients with hormone receptor-positive BC. This observed benefit in OS could be attributed to the higher percentage of patients with hormone receptor-positive BC in this study compared to those with triple-negative BC, as hormone receptor-positive BC tends to have better OS outcomes.

Several limitations must be considered when evaluating these results. First, this study was a retrospective, single-institution analysis, which may introduce selection and referral bias and limit generalizability. Second, differences in treatment setting, including patients treated within clinical trials and those treated in routine clinical practice, may have influenced outcomes. Third, the small sample size could also have obscured statistically significant differences. Fourth, the unavailability of toxicity data due to missing information is a notable limitation. Finally, the use of different PARPi and treatment regimens, including as single agents or in combination with chemotherapy, may have contributed to treatment heterogeneity and influenced clinical outcomes.

## 5. Conclusions

Our study provides valuable data on the use of PARPi in patients with advanced BC and germline *BRCA* PVs within the standard of care. These findings underscore the potential benefits of early PARPi therapy and the importance of considering patient-specific factors in treatment decisions. Further studies, including those on predictive markers of response and resistance, as well as sequencing with platinum drugs, are warranted to validate these findings in larger cohorts and to continue improving treatment guidelines for this challenging clinical situation.

## Figures and Tables

**Table 1:** Patient demographics and baseline disease characteristics (*n* = 107).

Characteristics	n (%)
**Age at Diagnosis, median (range)**	38 (23, 73)
**Gender**	
Male	2 (1.9)
Female	105 (98.1)
**Race**	
Asian	5 (4.7)
Black	11 (10.3)
White	76 (71.0)
Other	11 (10.3)
Unknown	4 (3.7)
***BRCA*** **Mutation Status**	
*BRCA1+*	48 (44.9)
*BRCA2+*	59 (55.1)
**Triple negative**	
Yes	42 (39.3)
No	65 (60.7)
**De novo** ** metastatic cancer (stage IV)**	
Yes	19 (17.8)
No	88 (82.2)
**Metastatic Sites before PARPi initiation ^a,^** ^†^	
Brain	9 (8.8%)
Bone	49 (48.0%)
Distant LNs	21 (20.6%)
Liver	35 (34.3%)
Lung	30 (29.4%)
Other distant sites *	68 (66.7%)
**Previous platinum use**	
Yes	24 (22.4)
No	82 (76.6)
Unknown	1 (0.9)
**BMI (kg/m^2^) ^b^**	
Median (Range)	25.9 (14.8, 61.2)
<18.5	4 (3.8)
18.5–24.9	43 (41.0)
25.0–29.9	30 (28.6)
≥30.0	28 (26.7)
**Systemic treatment before PARPi**	
Yes	62 (57.9)
No	45 (42.1)
**Lines of systemic treatment before PARPi ^c^**	
1	26 (41.9)
2	15 (24.2)
3	6 (9.7)
4	5 (8.1)
5	4 (6.5)
6	4 (6.5)
8	2 (3.2)
Median (Range)	2 (1, 8)
**PARPi drug type**	
Olaparib	52 (48.6)
Talazoparib	31 (29.0)
Veliparib	21 (19.6)
Other	3 (2.8)
**PARPi treatment type**	
Research	50 (46.7)
Standard of practice	48 (44.9)
Both	9 (8.4)
**Survival status**	
Alive/NED	8 (7.5)
Alive/WD	40 (37.4)
Dead/WD	59 (55.1)
**Follow-up time (months), median (range)**	16 (0, 127)

^a^ Data included for 102 patients. ^b^ Data included for 105 patients. ^c^ Data included for 62 patients. * Other distant sites included pleura, peritoneum/omentum, skin/chest wall, soft tissue/muscle, and other less common visceral sites. ^†^ Patients may have had multiple metastatic sites. Abbreviations: *BRCA*, breast cancer gene; BMI, body mass index; LNs, lymph nodes; NED, no evidence of disease; PARPi, PARP inhibitors; WD, with disease.

**Table 2:** Response to PARPi in Patients Who Received ≥ 2 Cycles of PARPi (*n* = 91).

	N (%)								
Subgroup	CR	PR	SD	PD	ORR (95% CI)	DCR (95% CI)	By Response	N	DoR, Median (Range)
All patients	4 (4.4)	53 (58.2)	18 (19.8)	16 (17.6)	62.6% (51.9–72.6%)	82.4% (73.0–89.6%)	Disease control	60	7.2 (1.4–96.2)
							Objective response	45	7.0 (2.1–96.2)
Platinum- naïve	4 (5.6)	46 (64.8)	9 (12.7)	12 (16.9)	70.4% (58.4–80.7%)	83.1% (72.3–91.0%)	Disease control	44	7.1 (1.4–96.2)
							Objective response	38	7.4 (2.1–96.2)
Previous platinum-based treatment	0 (0.0)	6 (31.6)	9 (47.4)	4 (21.0)	31.6% (12.6–56.6%)	79.0% (54.4–94.0%)	Disease control	15	7.3 (2.3–27.3)
							Objective response	6	6.0 (2.3–27.3)
Platinum based treatment for advanced disease	2 (5.7)	18 (51.4)	7 (20.0)	8 (22.9)	57.1% (39.4–73.7%)	77.1% (59.9–89.6%)	Disease control	25	6.8 (2.1–96.2)
							Objective response	19	6.8 (2.1–96.2)
First-line PARPi	3 (7.0)	28 (65.1)	6 (13.9)	6 (13.9)	72.1% (56.3–84.7%)	86.1% (72.1–94.7%)	Disease control	26	7.3 (2.3–32.1)
							Objective response	22	7.1 (2.3–32.1)
≥1 lines before PARPi	1 (2.1)	25 (52.1)	12 (25.0)	10 (20.8)	54.2% (39.2–68.6%)	79.2% (65.0–89.5%)	Disease control	34	7.0 (1.4–96.2)
							Objective response	23	6.8 (2.1–96.2)
PARPi as standard of practice	2 (4.9)	23 (56.1)	11 (26.8)	5 (12.2)	61.0% (44.5–75.8%)	87.8% (73.8–95.9%)	Disease control	24	7.4 (1.4–32.1)
							Objective response	16	7.2 (2.7–32.1)
PARPi as research	2 (4.9)	24 (58.5)	5 (12.2)	10 (24.4)	63.4% (46.9–77.9%)	75.6% (59.7–87.6%)	Disease control	28	6.7 (2.1–96.2)
							Objective response	23	7.4 (2.1–96.2)

Abbreviations: CR, complete response; PR, partial response; SD, stable disease; PD, progressive disease; ORR, objective response rate (CR + PR); DCR, disease control rate (CR + PR + SD); DoR, duration of response; CI, confidence interval; PARPi, PARP inhibitors.

**Table 3:** Univariate Analysis of Progression-free Survival.

Cohort	Total	Events	Median (Months) (95% CI)	Hazard Ratio (95% CI)	*p*-Value
**All Patients**	105	84	9.0 (6.9–10.5)		
**Age**					
<38	48	34	10.5 (6.9–18.6)	Reference	
≥38	57	50	7.4 (5.7–10.1)	1.43 (0.92–2.21)	0.1106
**Race**					
White	74	62	9.7 (7.7–11.8)	Reference	
Black	11	7	6.2 (4.9–NE)	0.86 (0.39–1.88)	0.7043
Asian	5	3	24.9 (5.1–NE)	0.70 (0.22–2.24)	0.5503
Other	11	8	6.5 (2.9–NE)	1.33 (0.63–2.78)	0.4530
***BRCA*** ** Mutation Status**					
*BRCA1*	46	38	9.2 (7.4–11.7)	Reference	
*BRCA2*	59	46	7.3 (5.7–12.4)	1.08 (0.70–1.66)	0.7213
**Histological Type**					
Ductal	96	76	9.0 (6.9–10.5)	Reference	
Lobular	4	3	8.9 (5.1–NE)	0.84 (0.26–2.66)	0.7616
Mixed	4	4	9.2 (0.9–NE)	1.51 (0.55–4.17)	0.4227
Other	1	1	3.9 (NE–NE)	4.69 (0.63–34.95)	0.1318
**Hormone status**					
ER and/or PgR Positive	64	49	9.7 (7.0–12.6)	Reference	
Triple Negative	41	35	7.3 (5.1–10.5)	1.28 (0.82–1.97)	0.2741
**HER2/neu**					
Negative	101	81	8.6 (6.3–10.5)	Reference	
Positive	3	3	14.5 (0.9–NE)	0.99 (0.31–3.14)	0.9833
**BMI (kg/m^2^)**					
<25	47	40	8.5 (6.2–11.7)	Reference	
25.0–29.9	29	23	9.0 (5.7–18.6)	0.92 (0.55–1.54)	0.7548
≥30.0	28	20	8.6 (4.1–36.2)	0.76 (0.44–1.30)	0.3112
**BMI (kg/m^2^)**					
<25.9	50	42	8.1 (6.3–11.3)	Reference	
≥25.9	54	41	9.0 (5.7–18.6)	0.81 (0.52–1.25)	0.3388
**Previous Therapy for** **Advanced Breast Cancer**					
No (first line PARPi use)	44	32	11.3 (8.5–24.9)	Reference	
Yes (≥1 line before PARPi use)	61	52	6.9 (5.7–10.3)	1.65 (1.06–2.56)	**0.0274**
**Previous Platinum Use**					
No	81	62	9.2 (6.9–11.8)	Reference	
Yes	23	21	7.4 (4.1–12.4)	1.61 (0.97–2.66)	0.0651
**PARPi Drug Type**					
Olaparib	50	40	9.2 (7.0–10.5)	Reference	
Talazoparib	31	23	9.0 (5.0–33.6)	0.83 (0.50–1.39)	0.4801
Veliparib	21	18	10.5 (5.7–29.4)	0.79 (0.45–1.38)	0.4010
Other	3	3	2.0 (0.9–NE)	9.12 (2.59–32.04)	**0.0006**
**PARPi Treatment Type**					
Standard of Care	46	31	10.3 (8.6–14.5)	Reference	
Research	50	45	5.7 (4.7–9.2)	1.59 (1.00–2.52)	**0.0478**
Both	9	8	9.8 (7.0–NE)	1.25 (0.57–2.72)	0.5745
**Brain Metastasis**					
No	97	76	9.0 (6.3–10.5)	Reference	
Yes	8	8	9.0 (6.2–NE)	1.29 (0.62–2.68)	0.4964
**Bone Metastasis**					
No	57	41	10.5 (7.4–16.7)	Reference	
Yes	48	43	6.9 (5.2–9.7)	1.72 (1.11–2.65)	**0.0144**
**Distant LN Metastasis**					
No	85	66	9.0 (6.9–10.5)	Reference	
Yes	20	18	7.4 (5.7–30.8)	1.06 (0.63–1.78)	0.8323
**Lung Metastasis**					
No	75	57	9.8 (8.1–14.0)	Reference	
Yes	30	27	5.3 (4.1–10.1)	1.76 (1.11–2.78)	**0.0166**
**Liver Metastasis**					
No	70	55	10.1 (7.4–11.7)	Reference	
Yes	35	29	6.1 (5.0–11.8)	1.34 (0.85–2.11)	0.2020
**Other Distant Sites** **Metastasis**					
No	33	27	10.5 (9.7–23.5)	Reference	
Yes	67	57	6.1 (5.1–8.5)	1.60 (1.01–2.53)	**0.0466**

Abbreviations: *BRCA*, breast cancer gene; BMI, body mass index; CI, confidence interval; ER, estrogen receptor; HER2, human epidermal growth factor receptor 2; LN, lymph node; NE = Not Estimable; PARPi, PARP inhibitors; PgR, progesterone receptor.

**Table 4:** Univariate Analysis of Overall Survival.

Cohort	Total	Deaths	Median (Months) (95% CI)	Hazard Ratio (95% CI)	*p*-Value
**All Patients**	107	59	25.8 (18.7–31.5)		
**Age**					
<38	49	28	20.1 (15.4–33.2)	Reference	
≥38	58	31	30.0 (19.1–61.5)	0.84 (0.50–1.40)	0.4939
**Race**					
White	76	44	25.8 (18.7–33.2)	Reference	
Black	11	5	19.4 (9.0–NE)	1.24 (0.49–3.16)	0.6469
Asian	5	2	31.5 (5.1–NE)	0.58 (0.14–2.40)	0.4509
Other	11	4	46.9 (15.4–NE)	0.80 (0.29–2.24)	0.6765
***BRCA*** ** Mutation Status**					
*BRCA1*	48	29	23.3 (16.4–64.0)	Reference	
*BRCA2*	59	30	27.4 (18.3–46.9)	1.02 (0.61–1.70)	0.9387
**Histological Type**					
Ductal	97	54	25.8 (19.1–33.2)	Reference	
Lobular	4	2	NE (5.2–NE)	1.11 (0.27–4.56)	0.8881
Mixed	5	2	NE (7.4–NE)	1.30 (0.31–5.41)	0.7181
Other	1	1	5.4 (NE–NE)	17.19 (2.06–143.19)	**0.0085**
**Hormone status**					
ER and/or PgR Positive	65	30	30.0 (25.8–NE)	Reference	
Triple Negative	42	29	16.4 (14.5–28.5)	1.97 (1.17–3.31)	**0.0107**
**HER2/neu**					
Negative	103	59	23.6 (18.3–30.9)	Reference	
Positive	3	0	NE (NE–NE)	0.00 (0.00–0.00)	0.9884
**BMI (kg/m^2^)**					
<25	47	27	23.5 (16.4–61.5)	Reference	
25.0–29.9	30	17	26.8 (15.4–NE)	0.93 (0.50–1.71)	0.8125
≥30.0	28	14	31.5 (19.1–NE)	0.92 (0.48–1.77)	0.8052
**BMI (kg/m^2^)**					
<25.9	51	28	23.5 (16.4–64.0)	Reference	
≥25.9	54	30	28.5 (19.1–46.9)	0.96 (0.57–1.62)	0.8907
**Previous Therapy for** **Advanced Breast Cancer**					
No (first line PARPi use)	45	23	30.9 (23.3–NE)	Reference	
Yes (≥1 line before PARPi use)	62	36	18.7 (14.7–30.0)	1.49 (0.88–2.53)	0.1352
**Previous Platinum Use**					
No	82	46	27.4 (19.4–36.5)	Reference	
Yes	24	12	19.1 (15.4–NE)	1.16 (0.61–2.20)	0.6462
**PARPi Drug Type**					
Olaparib	52	25	25.8 (19.1–36.5)	Reference	
Talazoparib	31	17	30.0 (12.7–NE)	1.21 (0.65–2.25)	0.5463
Veliparib	21	17	19.4 (12.0–61.5)	1.19 (0.63–2.24)	0.5935
Other	3	0	NE (NE–NE)	0.00 (0.00–0.00)	0.9874
**PARPi Treatment Type**					
Standard of Care	48	18	41.6 (19.1–NE)	Reference	
Research	50	37	19.4 (14.9–30.9)	1.43 (0.81–2.54)	0.2217
Both	9	4	36.5 (18.3–NE)	0.71 (0.24–2.10)	0.5335
**Brain Metastasis**					
No	98	51	28.2 (19.4–41.6)	Reference	
Yes	9	8	16.4 (9.0–NE)	2.48 (1.16–5.32)	**0.0196**
**Bone Metastasis**					
No	58	30	26.8 (20.1–NE)	Reference	
Yes	49	29	18.3 (14.5–41.6)	1.55 (0.93–2.58)	0.0955
**Distant LN Metastasis**					
No	86	46	25.8 (18.3–33.2)	Reference	
Yes	21	13	28.5 (17.8–NE)	0.95 (0.51–1.75)	0.8595
**Lung Metastasis**					
No	77	36	27.4 (23.3–61.5)	Reference	
Yes	30	23	15.4 (13.2–36.5)	1.91 (1.13–3.23)	**0.0159**
**Liver Metastasis**					
No	72	37	27.4 (18.7–46.9)	Reference	
Yes	35	22	23.6 (13.2–64.0)	1.23 (0.73–2.09)	0.4411
**Other Distant Sites** **Metastasis**					
No	34	19	28.2 (19.1–NE)	Reference	
Yes	68	40	20.1 (14.7–41.6)	1.43 (0.83–2.48)	0.1988

Abbreviations: *BRCA*, breast cancer gene; BMI, body mass index; CI, confidence interval; ER, estrogen receptor; HER2, human epidermal growth factor receptor 2; LN, lymph node; NE = Not Estimable; PARPi, PARP inhibitors; PgR, progesterone receptor.

## Data Availability

The datasets generated and/or analyzed in this study are not publicly available as the database is actively updated and is the list of active and prior patients receiving care at MD Anderson Cancer Center.

## Supplementary Materials

The following supporting information can be downloaded at: https://www.mdpi.com/article/10.3390/cancers18081258/s1, Table S1: Additional baseline demographic and clinical characteristics of the study cohort (*n*=107); Table S2: Baseline Demographic and Clinical Characteristics by Treatment Response Among Patients Receiving ≥2 Cycles of PARP Inhibitors; Figure S1: Progression-free Survival by Previous Therapy for Advanced Breast Cancer; Figure S2: Progression-free Survival by PARPi Treatment Type; Figure S3: Progression-free Survival by Bone Metastasis; Figure S4: Progression-free Survival by Lung Metastasis; Figure S5: Overall Survival by Triple Negative Status; Figure S6: Overall Survival by Brain Metastasis; Figure S7: Overall Survival by Lung Metastasis.

## Abbreviations

The following abbreviations are used in this manuscript:

ASCO	American Society of Clinical Oncology
BC	Breast cancer
BMI	Body mass index
*BRCA*	Breast cancer gene
CAP	College of American Pathologists
CI	Confidence interval
CR CT	Complete response Computed tomography
DCR	Disease control rate
DNA	Deoxyribonucleic acid
DoR	Duration of response
ER	Estrogen receptor
FDA	United States Food and Drug Administration
HER2	Human epidermal growth factor receptor 2
HR	Hazard ratio
LN/LNs	Lymph node(s)
NA	Not available
NED	No evidence of disease
ORR	Objective response rate
OS	Overall survival
PARP	Poly (ADP-ribose) polymerase
PARPi	Poly (ADP-ribose) polymerase inhibitors
PD	Progressive disease
PFS	Progression-free survival
PgR	Progesterone receptor
PR	Partial response
PV	Pathogenic variant
RECIST	Response Evaluation Criteria in Solid Tumors
SD	Stable disease
