# Supplementary material for: Characteristics and Clinical Outcomes of BRCA Germline Mutation Carriers with Advanced Breast Cancer Treated with PARP (Poly ADP-Ribose Polymerase) Inhibitors: A Single-Institution Experience
Source: Cancers (Basel). 2026 Apr 16;18(8):1258. doi: 10.3390/cancers18081258 (PMC13114508; doi:10.3390/cancers18081258)
Supplement: Supplementary file 1 [file cancers-18-01258-s001.zip › cancers-4212392-supplementary.pdf]

cancers

MDPI

Article

Characteristics and Clinical Outcomes of BRCA Germline Mutation Carriers with Advanced Breast Cancer Treated with PARP (Poly ADP-Ribose Polymerase) Inhibitors: A Single-Institution Experience †

Fatma Nihan Akkoc Mustafayev [1], Elena Fountzilas [2], Mark F. Munsell [3], Rachel M. Layman [1], Clinton Yam [1], Angelica M. Gutierrez [1], Constance T. Albarracin [4], Zamal Ahmed [5], Katharina Schlacher [6], John A. Tainer [5] and Banu K. Arun [1,*]

1 Department of Breast Medical Oncology, The University of Texas MD Anderson Cancer Center, Houston, Texas 77030, USA; nihanakkoc88@gmail.com (F.N.A.M.)
2 Department of Medical Oncology, St Luke's Clinic, Thessaloniki, Greece
3 Department of Biostatistics, The University of Texas MD Anderson Cancer Center, Houston, Texas 77030, USA
4 Department of Pathology, The University of Texas MD Anderson Cancer Center, Houston, Texas 77030, USA
5 Department of Molecular and Cellular Oncology, The University of Texas MD Anderson Cancer Center, Houston, Texas 77030, USA
6 Department of Cancer Biology, The University of Texas MD Anderson Cancer Center, Houston, Texas 77030, USA
* Correspondence: barun@mdanderson.org; Tel.: +1 (713) 792-2817; Fax: +1 (713) 794-4385

† This manuscript is an extended version of our preliminary findings presented at the 2023 ASCO Annual Meeting: Mustafayev FNA, Munsell M, Gutierrez-Barrera AM, Yam C, Layman RM, Arun B. Characteristics and clinical outcome of BRCA germline mutation carriers with advanced breast cancer treated with PARP (poly ADP-ribose polymerase) inhibitors: A single institution experience. J Clin Oncol. 2023;41(16_suppl):10587. doi: 10.1200/JCO.2023.41.16_suppl.10587.

Supplementary Table S1. Additional baseline demographic and clinical characteristics of the study cohort (*n*=107).

Characteristics	*n* (%)
**Family history of breast cancer and/or ovarian cancer**	
Yes	89 (83.2)
No	18 (16.8)
**Prophylactic mastectomy**	
Yes	68 (63.6)
No	39 (36.4)
**Bilateral salpingo-oophorectomy**	
Yes	61 (57.0)
No	44 (41.1)
NA	2 (1.9)
**Menopausal status at diagnosis**	
Post-menopausal	27 (25.2)
Pre-menopausal	76 (71.0)
Unknown	2 (1.9)
NA	2 (1.9)
**Histological type**	
Ductal	97 (90.7)

Characteristics	n (%)
Lobular	4 (3.7)
Mixed	5 (4.7)
Other	1 (0.9)
**ER status**	
Negative	46 (43.0)
Positive	60 (56.1)
Unknown	1 (0.9)
**PgR status**	
Negative	55 (51.4)
Positive	50 (46.7)
Unknown	2 (1.9)
**HER2/neu**	
Negative	103 (96.3)
Positive	3 (2.8)
Unknown	1 (0.9)
**Chemotherapy**	
Adjuvant	29 (27.1)
Neoadjuvant	31 (29.0)
Both	25 (23.4)
None	22 (20.5)
**Hormone therapy**	
Adjuvant	45 (42.1)
Neoadjuvant	1 (0.9)
Both	1 (0.9)
None	60 (56.1)
**Less than 12-month disease-free interval from initial diagnosis to advanced breast cancer**	
Yes	24 (22.4)
No	83 (77.6)
**Platinum-based neoadjuvant or adjuvant therapy**	
Yes	12 (11.2)
No	95 (88.8)
**Platinum-based chemotherapy for advanced disease**	
Yes	42 (39.2)
No	58 (54.2)
Unknown	2 (1.9)
NA	5 (4.7)

Abbreviations: ER, estrogen receptor; HER2, human epidermal growth factor receptor 2; NA, not available; PgR, progesterone receptor.

Supplementary Table S2. Baseline Demographic and Clinical Characteristics by Treatment Response Among Patients Receiving ≥2 Cycles of PARP Inhibitors.

	Response to PARPi			
**Characteristics**	PD+SD (*n*=34)	CR+PR (*n*=57)	Total (*n*=91)	P-value
**Age at Diagnosis**				0.5272[1]
n	34	57	91	
Median (range)	41 (25, 62)	38 (23, 73)	38 (23, 73)	
**Gender, *n* (%)**				0.5267[2]
Female	34 (100.0%)	55 (96.5%)	89 (97.8%)	

	Response to PARPi			
**Characteristics**	PD+SD (*n*=34)	CR+PR (*n*=57)	Total (*n*=91)	P-value
Male	0 (0.0%)	2 (3.5%)	2 (2.2%)	
**Race, *n* (%)**				0.4406[2]
White	25 (75.8%)	42 (76.4%)	67 (76.1%)	
Black	3 (9.1%)	6 (10.9%)	9 (10.2%)	
Asian	0 (0.0%)	3 (5.5%)	3 (3.4%)	
Other	5 (15.2%)	4 (7.3%)	9 (10.2%)	
Unknown	1	2	3	
***BRCA* Mutation Status, *n* (%)**				>0.9999[2]
BRCA1	16 (47.1%)	26 (45.6%)	42 (46.2%)	
BRCA2	18 (52.9%)	31 (54.4%)	49 (53.8%)	
**Family history of breast cancer and/or ovarian cancer, *n* (%)**				0.5602[2]
Yes	27 (79.4%)	49 (86.0%)	76 (83.5%)	
No	7 (20.6%)	8 (14.0%)	15 (16.5%)	
**Prophylactic mastectomy, *n* (%)**				0.3720[2]
Yes	19 (55.9%)	38 (66.7%)	57 (62.6%)	
No	15 (44.1%)	19 (33.3%)	34 (37.4%)	
**Bilateral Salpingo-oophorectomy, *n* (%)**				0.0765[2]
Yes	16 (47.1%)	37 (67.3%)	53 (59.6%)	
No	18 (52.9%)	18 (32.7%)	36 (40.4%)	
NA	0	2	2	
**Menopausal status at diagnosis, *n* (%)**				0.3183[2]
Premenopausal	22 (66.7%)	42 (77.8%)	64 (73.6%)	
Postmenopausal	11 (33.3%)	12 (22.2%)	23 (26.4%)	
Unknown	1	1	2	
NA	0	2	2	
**Histological type, *n* (%)**				0.1418[2]
Ductal	29 (85.3%)	55 (96.5%)	84 (92.3%)	
Lobular	2 (5.9%)	1 (1.8%)	3 (3.3%)	
Mixed	2 (5.9%)	1 (1.8%)	3 (3.3%)	
Other	1 (2.9%)	0 (0.0%)	1 (1.1%)	
**ER status, *n* (%)**				0.1963[2]
Negative	19 (55.9%)	23 (41.1%)	42 (46.7%)	
Positive	15 (44.1%)	33 (58.9%)	48 (53.3%)	
Unknown	0	1	1	
**PgR status, *n* (%)**				0.6626[2]
Negative	17 (51.5%)	32 (57.1%)	49 (55.1%)	
Positive	16 (48.5%)	24 (42.9%)	40 (44.9%)	
Unknown	1	1	2	
**HER2/neu, *n* (%)**				0.5267[2]
Negative	34 (100.0%)	55 (96.5%)	89 (97.8%)	
Positive	0 (0.0%)	2 (3.5%)	2 (2.2%)	
**Triple negative, *n* (%)**				0.2735[2]
Yes	17 (50.0%)	21 (36.8%)	38 (41.8%)	
No	17 (50.0%)	36 (63.2%)	53 (58.2%)	
**De novo metastatic cancer (Stage IV), *n* (%)**				0.5819[2]
Yes	5 (14.7%)	12 (21.1%)	17 (18.7%)	
No	29 (85.3%)	45 (78.9%)	74 (81.3%)	
**Metastatic sites before PARPi, *n* (%) †**				
Brain	3 (8.8%)	3 (5.3%)	6 (6.6%)	0.6675[2]

	Response to PARPi			
**Characteristics**	PD+SD (*n*=34)	CR+PR (*n*=57)	Total (*n*=91)	P-value
Bone	19 (55.9%)	22 (38.6%)	41 (45.1%)	0.1305[2]
Distant LN	4 (11.8%)	14 (24.6%)	18 (19.8%)	0.1785[2]
Lung	13 (38.2%)	12 (21.1%)	25 (27.5%)	0.0923[2]
Liver	9 (26.5%)	19 (33.3%)	28 (30.8%)	0.6394[2]
Other distant sites*	22 (64.7%)	36 (66.7%)	58 (65.9%)	>0.9999[2]
NA	0	3	3	
**Chemotherapy, *n* (%)**				0.5823[2]
Adjuvant	7 (20.6%)	17 (29.8%)	24 (26.4%)	
Neoadjuvant	12 (35.3%)	13 (22.8%)	25 (27.5%)	
Both	7 (20.6%)	14 (24.6%)	21 (23.1%)	
None	8 (23.5%)	13 (22.8%)	21 (23.1%)	
**Hormone therapy, *n* (%)**				0.5526[2]
Adjuvant	12 (35.3%)	24 (42.1%)	36 (39.6%)	
Neoadjuvant	1 (2.9%)	0 (0.0%)	1 (1.1%)	
Both	0 (0.0%)	1 (1.8%)	1 (1.1%)	
None	21 (61.8%)	32 (56.1%)	53 (58.2%)	
**Less than 12-month disease-free interval from initial diagnosis to advanced breast cancer, *n* (%)**				0.7984[2]
Yes	8 (23.5%)	12 (21.1%)	20 (22.0%)	
No	26 (76.5%)	45 (78.9%)	71 (78.0%)	
**Previous platinum Use, *n* (%)**				**0.0031[2]**
Yes	13 (38.2%)	6 (10.7%)	19 (21.1%)	
No	21 (61.8%)	50 (89.3%)	71 (78.9%)	
Unknown	0	1	1	
**Platinum-based neoadjuvant or adjuvant therapy, n (%)**				**0.0362[2]**
Yes	7 (20.6%)	3 (5.3%)	10 (11.0%)	
No	27 (79.4%)	54 (94.7%)	81 (89.0%)	
**Platinum-based chemotherapy for advanced disease, *n* (%)**				0.6563[2]
Yes	15 (45.5%)	21 (39.6%)	36 (41.9%)	
No	18 (54.5%)	32 (60.4%)	50 (58.1%)	
Unknown	1	4	5	
**BMI (kg/m^2), *n* (%)**				0.7783[2]
< 18.5	0 (0.0%)	2 (3.5%)	2 (2.2%)	
18.5-24.9	13 (40.6%)	25 (43.9%)	38 (42.7%)	
25.0-29.9	9 (28.1%)	16 (28.1%)	25 (28.1%)	
≥ 30.0	10 (31.3%)	14 (24.6%)	24 (27.0%)	
Unknown	2	0	2	
**Systemic treatment before PARPi**, *n* (%)				0.0873[2]
No	12 (35.3%)	31 (54.4%)	43 (47.3%)	
Yes	22 (64.7%)	26 (45.6%)	48 (52.7%)	
**Lines of systemic treatment before PARPi**				0.1682[1]
n	22	26	48	
Median (range)	2 (1, 8)	2 (1, 6)	2 (1, 8)	
**PARPi Drug Type, *n* (%)**				0.1317[2]
Olaparib	20 (58.8%)	26 (45.6%)	46 (50.5%)	
Talazoparib	7 (20.6%)	19 (33.3%)	26 (28.6%)	
Veliparib	5 (14.7%)	12 (21.1%)	17 (18.7%)	

https://doi.org/10.3390/cancers18081258

**Characteristics**	**Response to PARPi**			
	PD+SD (*n*=34)	CR+PR (*n*=57)	Total (*n*=91)	P-value
Other	2 (5.9%)	0 (0.0%)	2 (2.2%)	
**PARPi treatment type, *n* (%)**				>0.9999[2]
Research	15 (44.1%)	26 (45.6%)	41 (45.1%)	
Standard of Care	16 (47.1%)	25 (43.9%)	41 (45.1%)	
Both	3 (8.8%)	6 (10.5%)	9 (9.9%)	
**Survival status, *n* (%)**				0.1125[2]
Alive/NED	0 (0.0%)	6 (10.5%)	6 (6.6%)	
Alive/WD	13 (38.2%)	24 (42.1%)	37 (40.7%)	
Dead/WD	21 (61.8%)	27 (47.4%)	48 (52.7%)	
**Follow-up time (months)**				**0.0044[1]**
n	34	57	91	
Median (range)	15 (4, 53)	20 (4, 127)	18 (4, 127)	

[1]Wilcoxon rank sum p-value; [2]Fisher Exact p-value

† Patients may have had multiple metastatic sites.

*Other distant sites included pleura, peritoneum/omentum, skin/chest wall, soft tissue/muscle, and other less common visceral sites.

Abbreviations: *BRCA*, breast cancer gene; BMI, body mass index; CR, complete response; ER, estrogen receptor; HER2, human epidermal growth factor receptor 2; LNs, lymph nodes; NA, not applicable; NED, no evidence of disease; PARPi, PARP inhibitors; PD, progressive disease; PgR, progesterone receptor; SD, stable disease; WD, with disease.

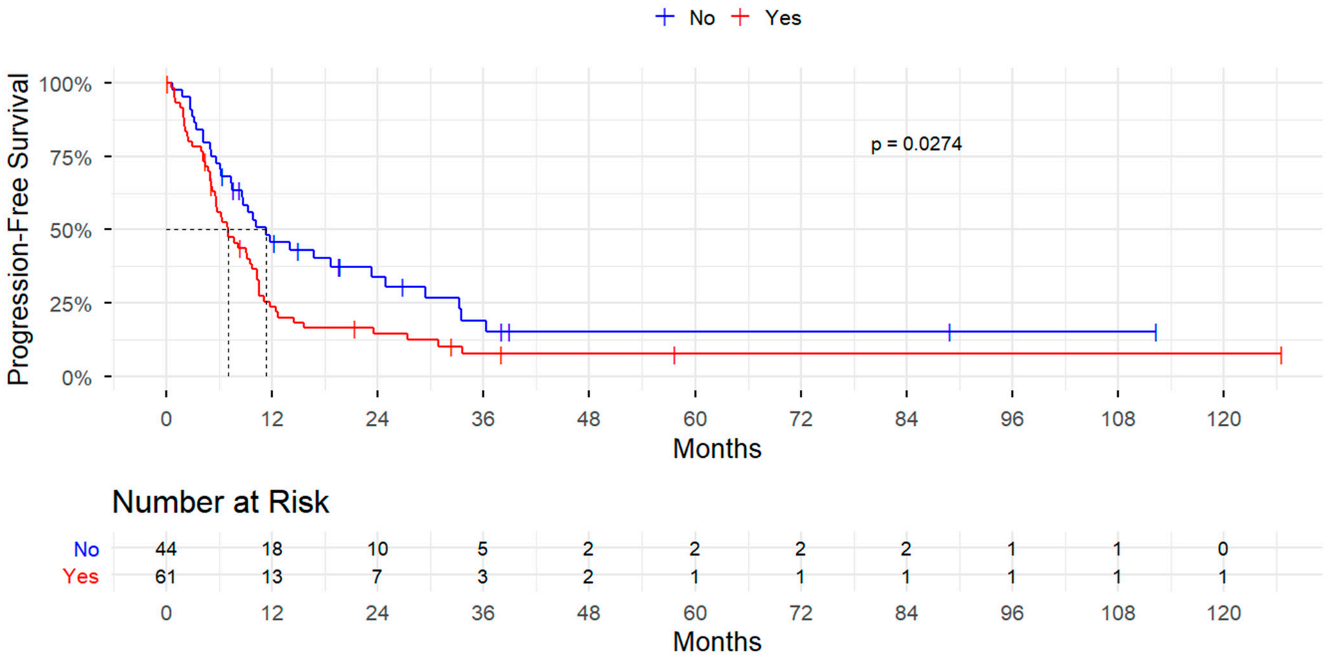

Supplementary Figure S1. Progression-free Survival by Previous Therapy for Advanced Breast Cancer.

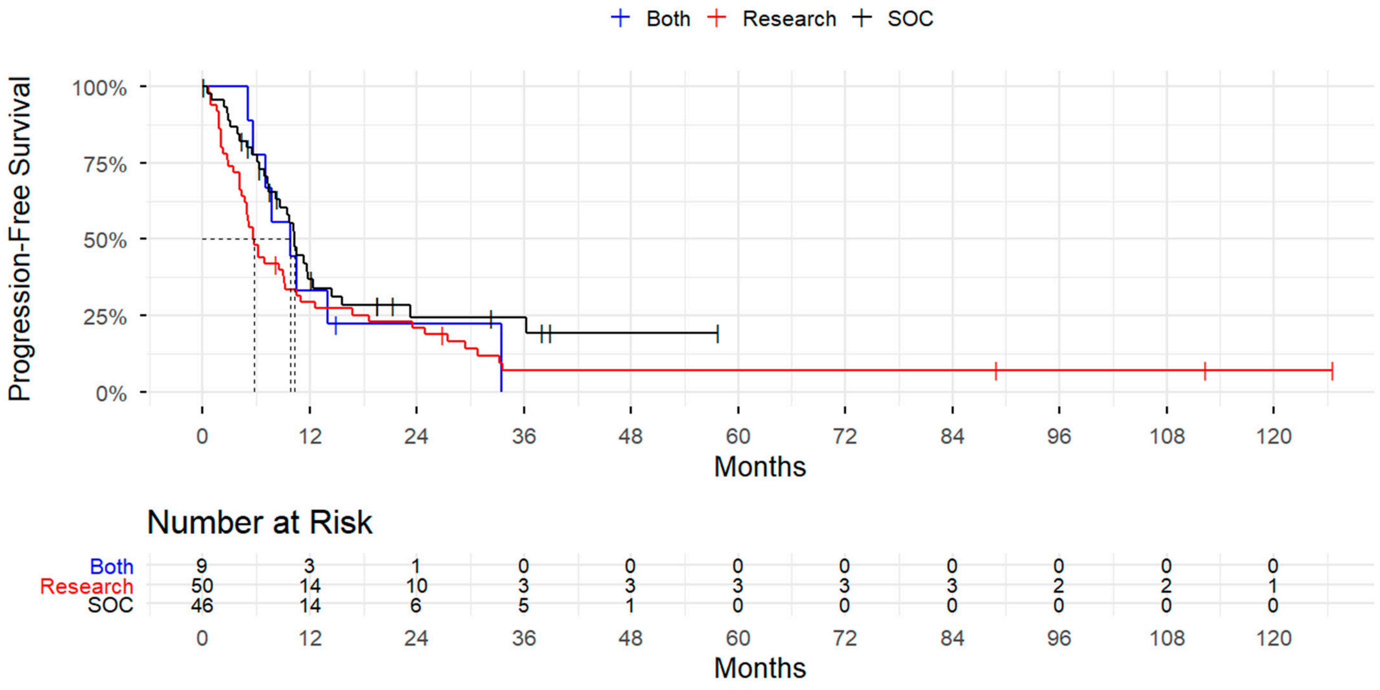

Supplementary Figure S2. Progression-free Survival by PARPi Treatment Type.

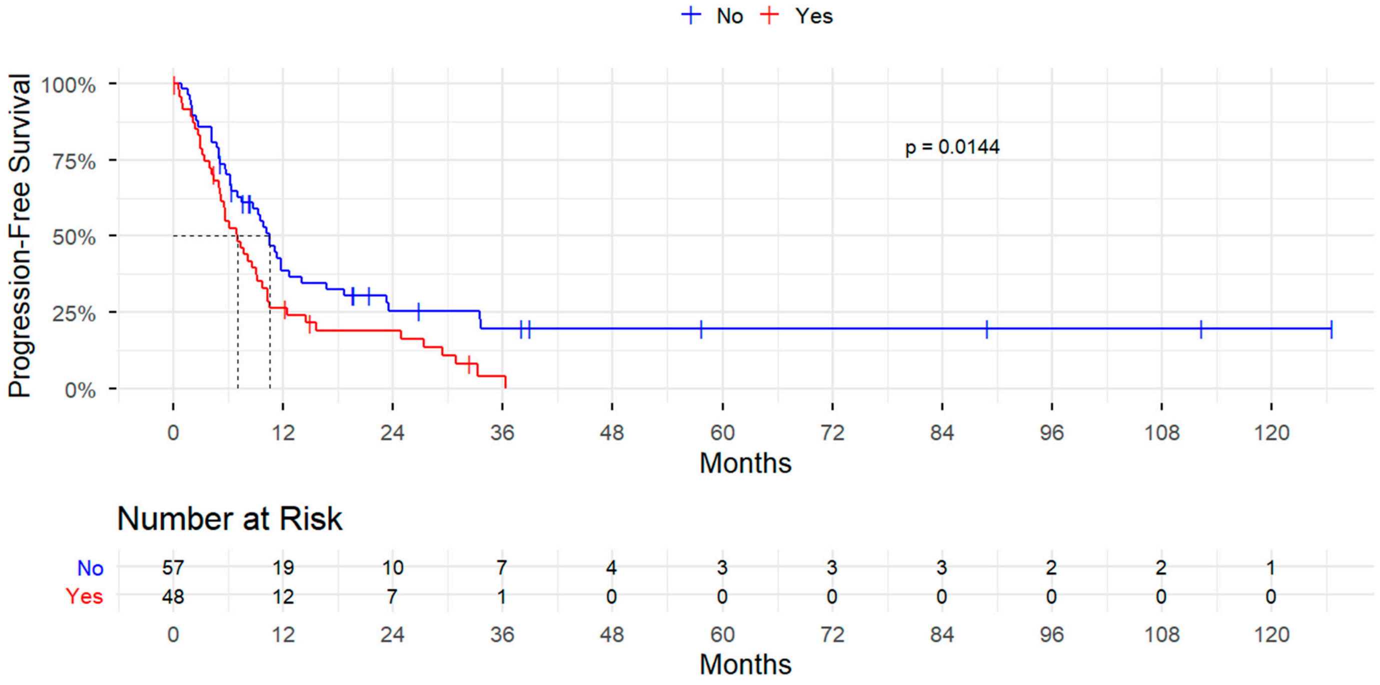

Supplementary Figure S3. Progression-free Survival by Bone Metastasis.

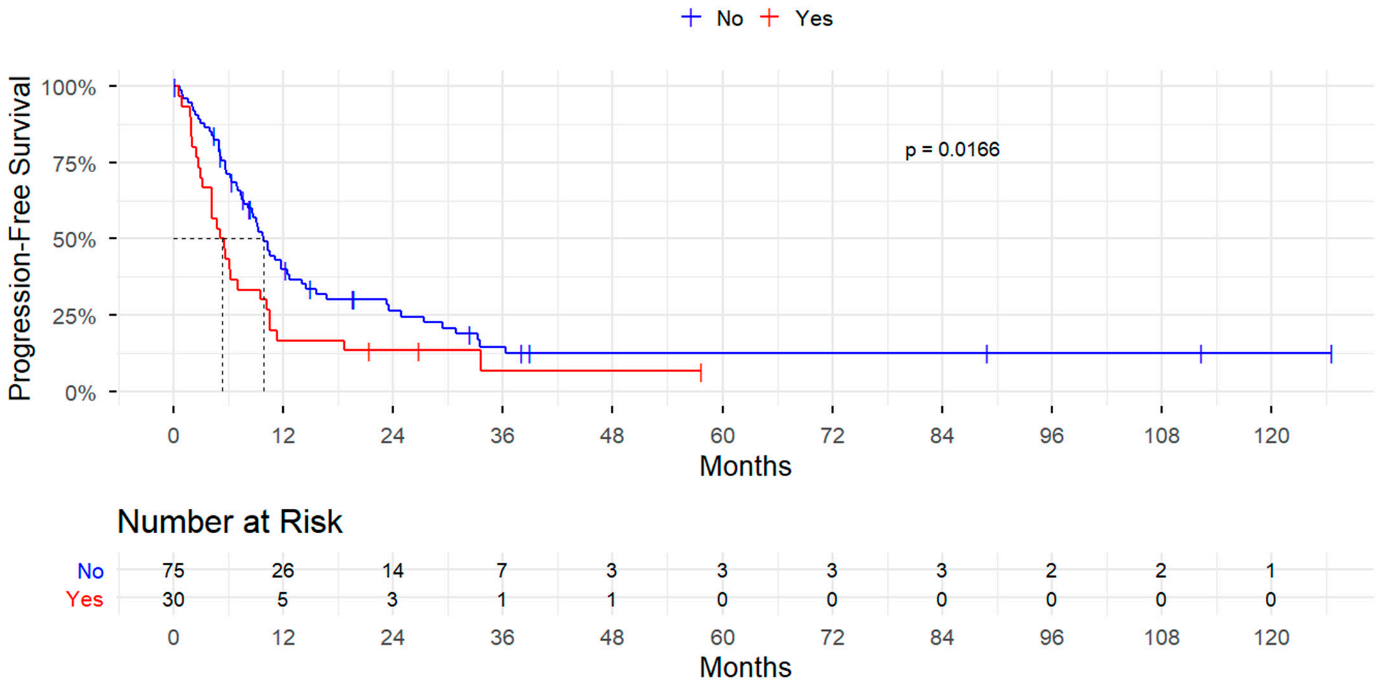

Supplementary Figure S4. Progression-free Survival by Lung Metastasis.

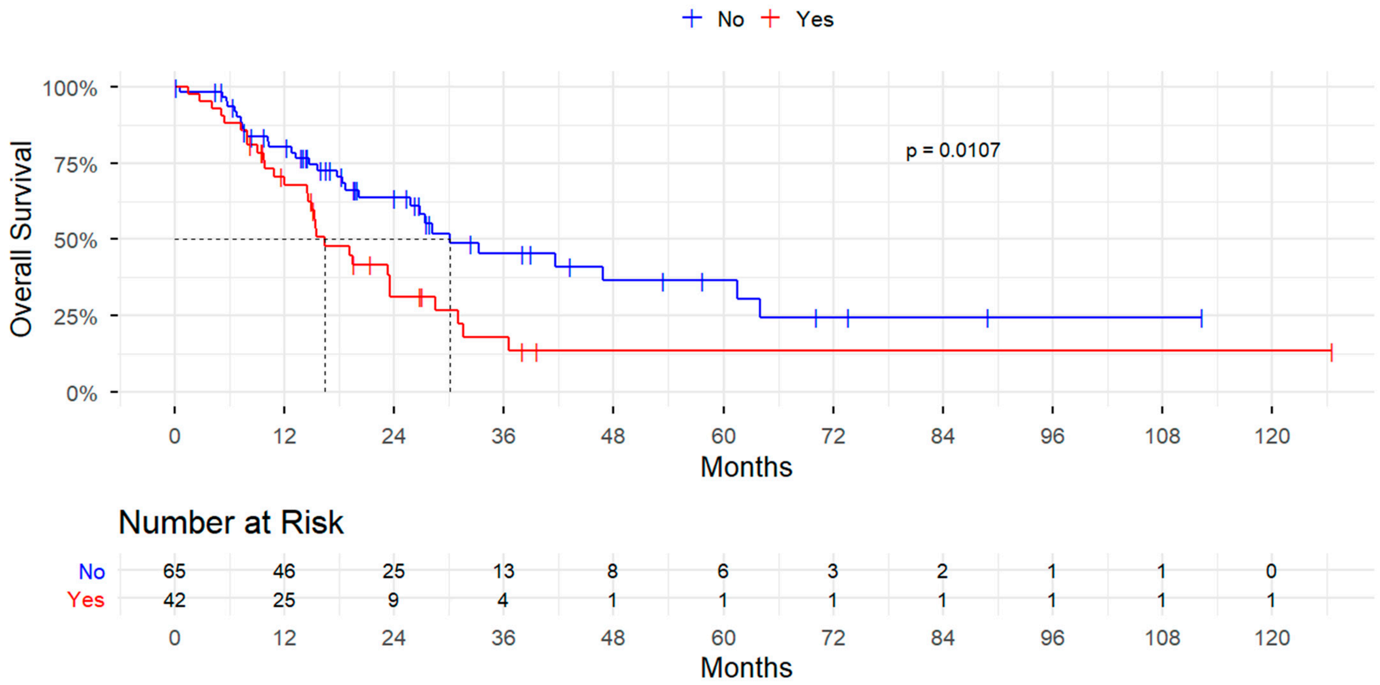

Supplementary Figure S5. Overall Survival by Triple Negative Status.

https://doi.org/10.3390/cancers18081258

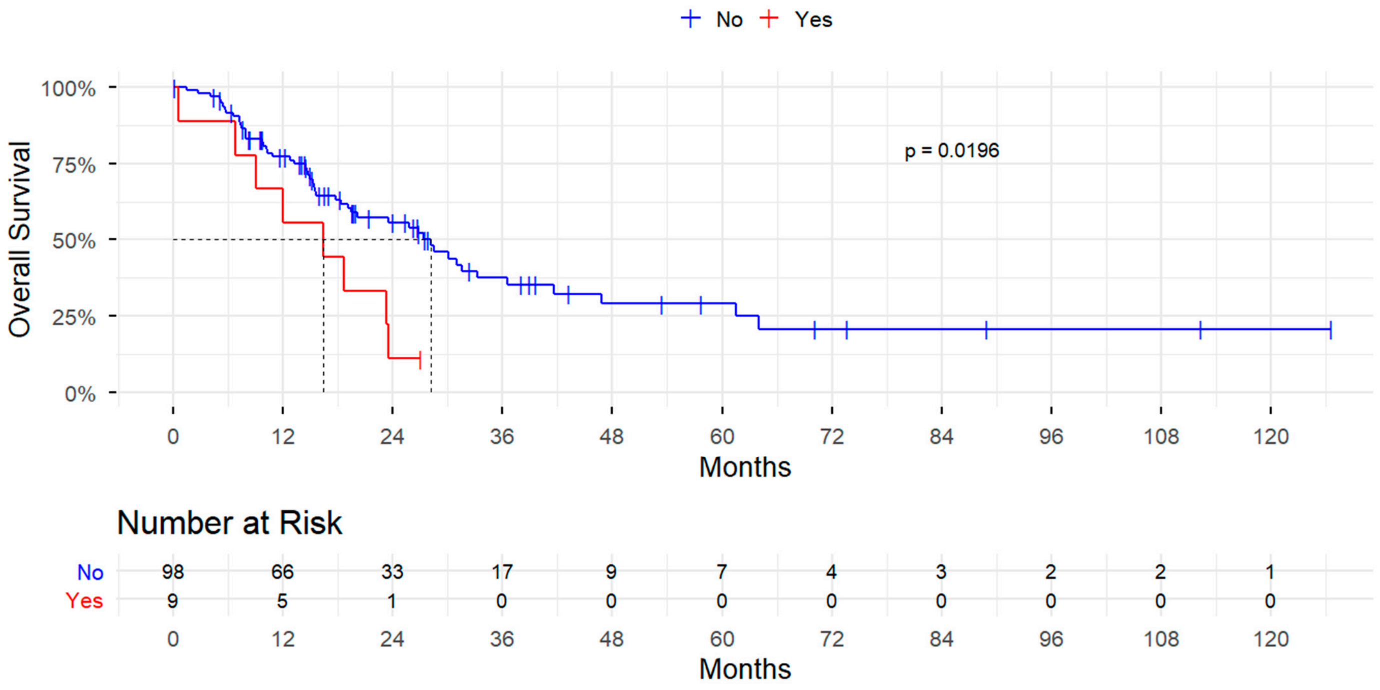

Supplementary Figure S6. Overall Survival by Brain Metastasis.

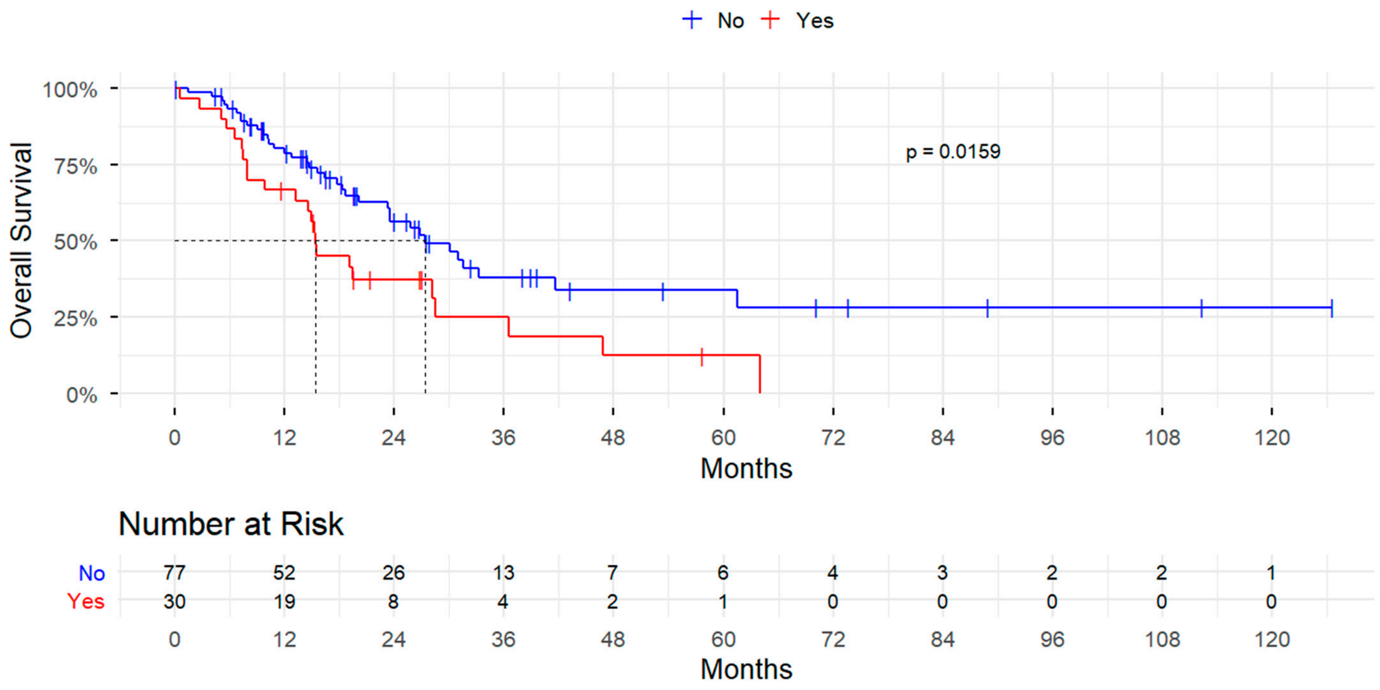

Supplementary Figure S7. Overall Survival by Lung Metastasis.

https://doi.org/10.3390/cancers18081258
